# Next-generation sequencing and FISH studies reveal the appearance of gene mutations and chromosomal abnormalities in hematopoietic progenitors in chronic lymphocytic leukemia

**DOI:** 10.1186/s13045-017-0450-y

**Published:** 2017-04-11

**Authors:** Miguel Quijada-Álamo, María Hernández-Sánchez, Cristina Robledo, Jesús-María Hernández-Sánchez, Rocío Benito, Adrián Montaño, Ana E. Rodríguez-Vicente, Dalia Quwaider, Ana-África Martín, María García-Álvarez, María Jesús Vidal-Manceñido, Gonzalo Ferrer-Garrido, María-Pilar Delgado-Beltrán, Josefina Galende, Juan-Nicolás Rodríguez, Guillermo Martín-Núñez, José-María Alonso, Alfonso García de Coca, José A. Queizán, Magdalena Sierra, Carlos Aguilar, Alexander Kohlmann, José-Ángel Hernández, Marcos González, Jesús-María Hernández-Rivas

**Affiliations:** 1 Servicio de Hematología & IBSAL, IBMCC, CIC Universidad de Salamanca-CSIC, Hospital Universitario, Salamanca, Spain; 2 Department of Molecular and Clinical Pharmacology, University of Liverpool, Liverpool, UK; 3 Servicio de Hematología, Hospital Virgen Blanca, León, Spain; 4 Servicio de Hematología, Hospital Miguel Servet, Zaragoza, Spain; 5 Servicio de Hematología, Hospital del Bierzo, Ponferrada, León, Spain; 6 Servicio de Hematología, Hospital Juan Ramón Jiménez, Huelva, Spain; 7 Servicio de Hematología, Hospital Virgen del Puerto, Plasencia, Cáceres, Spain; 8 Servicio de Hematología, Hospital Río Carrión, Palencia, Spain; 9 Servicio de Hematología, Hospital Clínico, Valladolid, Spain; 10 Servicio de Hematología, Hospital General de Segovia, Segovia, Spain; 11 Servicio de Hematología, Hospital Virgen de la Concha, Zamora, Spain; 12 Servicio de Hematología, Hospital Santa Bárbara, Soria, Spain; 13 MLL Munich, Munich, Germany; 14 AstraZeneca, Personalized Healthcare and Biomarkers, Innovative Medicines, Cambridge, UK; 15 Servicio de Hematología, Hospital Universitario Infanta Leonor, Universidad Complutense de Madrid, Madrid, Spain; 16 IBMCC, CIC Universidad de Salamanca-CSIC, Hospital Universitario de Salamanca, Paseo de San Vicente s/n, 37007 Salamanca, Spain

**Keywords:** Chronic lymphocytic leukemia, Next-generation sequencing, Hematopoietic progenitors, Mutation, FISH, Chromosomal abnormality

## Abstract

**Background:**

Chronic lymphocytic leukemia (CLL) is a highly genetically heterogeneous disease. Although CLL has been traditionally considered as a mature B cell leukemia, few independent studies have shown that the genetic alterations may appear in CD34+ hematopoietic progenitors. However, the presence of both chromosomal aberrations and gene mutations in CD34+ cells from the same patients has not been explored.

**Methods:**

Amplicon-based deep next-generation sequencing (NGS) studies were carried out in magnetically activated-cell-sorting separated CD19+ mature B lymphocytes and CD34+ hematopoietic progenitors (*n* = 56) to study the mutational status of *TP53*, *NOTCH1*, *SF3B1*, *FBXW7*, *MYD88*, and *XPO1* genes. In addition, ultra-deep NGS was performed in a subset of seven patients to determine the presence of mutations in flow-sorted CD34+CD19− early hematopoietic progenitors. Fluorescence in situ hybridization (FISH) studies were performed in the CD34+ cells from nine patients of the cohort to examine the presence of cytogenetic abnormalities.

**Results:**

NGS studies revealed a total of 28 mutations in 24 CLL patients. Interestingly, 15 of them also showed the same mutations in their corresponding whole population of CD34+ progenitors. The majority of *NOTCH1* (7/9) and *XPO1* (4/4) mutations presented a similar mutational burden in both cell fractions; by contrast, mutations of *TP53* (2/2), *FBXW7* (2/2), and *SF3B1* (3/4) showed lower mutational allele frequencies, or even none, in the CD34+ cells compared with the CD19+ population. Ultra-deep NGS confirmed the presence of *FBXW7*, *MYD88*, *NOTCH1*, and *XPO1* mutations in the subpopulation of CD34+CD19− early hematopoietic progenitors (6/7). Furthermore, FISH studies showed the presence of 11q and 13q deletions (2/2 and 3/5, respectively) in CD34+ progenitors but the absence of *IGH* cytogenetic alterations (0/2) in the CD34+ cells. Combining all the results from NGS and FISH, a model of the appearance and expansion of genetic alterations in CLL was derived, suggesting that most of the genetic events appear on the hematopoietic progenitors, although these mutations could induce the beginning of tumoral cell expansion at different stage of B cell differentiation.

**Conclusions:**

Our study showed the presence of both gene mutations and chromosomal abnormalities in early hematopoietic progenitor cells from CLL patients.

**Electronic supplementary material:**

The online version of this article (doi:10.1186/s13045-017-0450-y) contains supplementary material, which is available to authorized users.

## Background

Chronic lymphocytic leukemia (CLL) is characterized by the clonal proliferation and accumulation of neoplastic B lymphocytes in the blood, bone marrow, lymph nodes, and spleen [1, 2]. Immunophenotype analysis of CLL cells shows expression of CD5 T cell antigen as well as CD19, CD20, and CD23 B cell surface antigens [3]. In molecular terms, CLL is defined by the presence of chromosomal abnormalities (11q-, +12, 13q-, 17p-) that play an important role in CLL prognosis [4]. The mutational status of the immunoglobulin heavy chain (*IGHV*) is also considered a prognostic marker in CLL [5, 6]. Recently, the development of next-generation sequencing (NGS) techniques has enabled mutations to be identified in novel target genes in CLL [7, 8], and mutations in some drivers such as *NOTCH1*, *SF3B1*, *TP53*, and *MYD88* genes have been shown to have a prognostic impact in CLL patients [9–11].

The cellular origin of this disease remains controversial [12–14]. Recent studies have reported that CLL pathogenesis may start at a previous maturational cell stage, or even in hematopoietic stem cells (HSCs). Fluorescence in situ hybridization (FISH) studies showed that +12 and 13q- abnormalities are present in CD34+CD19− cells, suggesting that these common chromosomal abnormalities could appear in HSCs [15, 16]. Interestingly, xenotransplantation studies reported that HSCs from CLL patients were able to reproduce the CLL phenotype in murine models [17]. In addition, CLL mutations may appear in HSCs, supporting the idea that CLL pathogenic events occur at an early stage of the hematopoietic process [18].

Taking the previous studies in this field into account, it is well known that chromosomal abnormalities as well as gene mutations are important events in CLL pathogenesis [19]. However, it is still not clear which genetic events are related with the origin of the disease and when these alterations occur and have a functional impact inducing tumoral cell expansion during B cell differentiation. For these reasons, in this study, chromosomal abnormalities and gene mutations in hematopoietic progenitors were analyzed, showing that the whole population of CD34+ progenitors, even at the level of CD34+CD19−, are already affected at genetic level in CLL patients. In particular, mutations of *FBXW7*, *MYD88*, *NOTCH1*, and *XPO1* as well as 11q and 13q deletions were detected in CD34+ progenitors. By contrast, the origin of *TP53* and *SF3B1* mutations and *IGH* alterations could take place at a later maturational stage. Apart from B lymphocytes, some of these genetic alterations were also observed in other mature cell fractions (T lymphocytes and monocytes) derived from HSCs. Integrating all these results, a pattern of appearance and expansion of these genetic events during B-CLL cell differentiation was suggested.

## Methods

### Patients

Samples were collected from the bone marrow (BM) of 56 CLL patients. CLL was diagnosed according to the World Health Organization (WHO) classification [20] and the National Cancer Institute (NCI) Working Group criteria [21]. A complete immunophenotypic analysis of all cases was carried out by flow cytometry. The main biological features of the CLL patients are summarized in Additional file 1: Table S1.

### Cell isolation and DNA extraction

Total CD34+ progenitor cells and CD19+ B cells were separately isolated from BM samples of CLL patients using magnetically activated cell sorting (MACS) CD34 and CD19 MicroBeads (Miltenyi Biotec, Bergisch Gladbach, Germany), respectively, according to the manufacturer’s instructions. The workflow followed consisted of three steps: first, the isolation of the whole population of CD34+ cells (including CD34+CD19− early progenitors and CD34+CD19+ pro-B cells) from the total BM mononuclear cells, followed by the selection of CD19+ cells from the CD34 negative cell fraction resultant from the first step. Cell purities were determined by flow cytometry, being greater than 90 and 98% for each CD34+ and CD19+ cell fractions, respectively.

In addition, fluorescence-activated cell sorting (FACS) (BD Biosciences, San Jose, CA, USA) was carried out in order to sort the specific subpopulation of CD34+CD19− cells as well as other mature cells such as CD19+ B lymphocytes, CD3+ T lymphocytes, and CD14+ monocytes, from peripheral blood (PB) samples in a second time point of the disease of seven CLL patients. Samples were stained with FITC anti-CD14 (Beckman Coulter), phycoerythrin (PE) anti-CD3 (Becton Dickinson), PE-Cy7 anti-CD19 (Immunostep S.L.), PerCP-Cy5.5 anti-CD45 (BioLegend), and allophycocyanin (APC) anti-CD34 (Becton Dickinson). Purities were greater than 98% in all cell fractions (Additional file 1: Figure S1).

Genomic DNA was extracted from the different cell populations by column-based purification (AllPrep DNA/RNA Mini Kit, Qiagen, Hilden, Germany) following the manufacturer’s instructions.

### Next-generation sequencing

NGS was performed in CD19+ B lymphocytes from all 56 CLL patients. Amplicon-based NGS was carried out on a GS Junior platform (454 Life Sciences, Branford, CT, USA) using the 454 Titanium Amplicon system (Roche Applied Science, Penzberg, Germany) [22] to investigate the mutational status of *TP53* (exons 4–11), *NOTCH1* (exons 33–34), *SF3B1* (exons 10–16), *FBXW7* (exons 8–12), *MYD88* (exons 4–5), and *XPO1* (exons 14–15) in CD19+ cells. The mutations identified in CD19+ cells were further analyzed in the corresponding whole population of CD34+ progenitors in order to determine whether the same mutations were present in an earlier step than B mature cells. Primer information, PCR conditions, and oligonucleotide design used in previous studies were adopted [23, 24]. The oligonucleotide was designed as part of the work of the IRON-II network. Sequencing data were obtained and analyzed using the GS Data Analysis Software package (Roche Applied Science, Penzberg, Germany) and the Sequence Pilot software for genetic analysis (JSI Medical Systems, Ettenheim, Germany). Mutations detected in more than 2% of bidirectional reads per amplicon in CD19+ cells and in more than 10% in CD34+ cells were accepted taking into account sequencing coverage (median 980 reads; coverage range 304–9387-fold) [25, 26] and MACS purities from each cell population (98% for CD19+ and 90% for CD34+ cells).

### Ultra-deep NGS

To define if the mutations appeared in the specific subpopulation of CD34+CD19− cells and other mature populations derived from the hematopoietic progenitors, mutated target regions were sequenced by ultra-deep NGS, using an Illumina platform, in flow-sorted CD34+CD19−, CD19+, CD3+, and CD14+ cell populations from seven CLL patients. NGS analysis was performed on MiSeq (Illumina, San Diego, CA, USA) using genomic DNA from peripheral blood flow-sorted CD19+ B lymphocytes, CD34+CD19− early progenitors, CD3+ T lymphocytes, and CD14+ monocytes. DNA was amplified using REPLI-g Mini Kit (Qiagen, Hilden, Germany). Target PCRs were performed using exon-specific primers (Additional file 1: Table S2). The experimental design and reaction conditions followed the manufacturer’s recommendations. Briefly, PCR products were purified with High Pure PCR Product Purification Kit (Roche Diagnostics, Mannheim, Germany) and quantified using Qubit dsDNA HS Assay Kit (Life Technologies, Waltham, MA, USA). The purified amplicons were pooled to a total amount of 50 ng. The indexed paired-end library was prepared with NEBNext Ultra II DNA Library Prep kit for Illumina (NEW ENGLAND BioLabs) and sequenced using MiSeq (median coverage 4399 reads; range 1491–8614-fold). In order to verify the accuracy of the variant allele frequency (VAF), non-amplified DNA was sequenced in all cases with available material, finding no differences comparing to the VAFs obtained with a previous step of whole-genome amplification.

In-house pipeline was performed to analyze sequencing data. Sequencing reads were aligned to the reference genome GRCh37/hg19 using BWA-0.7 [27]. The alignments were refined with tools of the GATK-3.5 suite [28], and the variants were called according to GATK Best Practice recommendations [29, 30]. Finally, ANNOVAR was used for annotations and prediction of functional consequences [31].

The variant detection was set at 2% taking into account the sequencing coverage and the purities from all sorted cell fractions (more than 98%). Mutations detected at low frequencies (< 15%) by ultra-deep NGS were also validated using 454 Titanium Amplicon System (Roche Applied Science, Penzberg, Germany) (median 1712 reads; coverage range 1277–2638-fold) [23, 24].

### Fluorescence in situ hybridization

Interphase FISH was carried out in B cells from 56 BM samples using commercially available probes: 11q22/*ATM*, 12p11.1-q11 (alpha satellite), 13q14, 14q32/*IGH*, and 17p13/*TP53* (Vysis/Abbott Co, Abbott Park, IL, USA). Dual-color FISH using differently labeled control and test probes was implemented following the methods previously described [32]. FISH was also performed in the CD34+ cells of a group of nine CLL patients to assess the presence of the cytogenetic alterations identified in the corresponding CD19+ cell fraction. Samples were placed in a Cytospin cytocentrifuge (Thermo Scientific, Waltham, MA, USA) to concentrate the low number of cells. Signal screening was performed in at least 200 cells with well-delineated fluorescence spots. According to our cut-off standards, a score ≥ 10% was considered positive in all cases.

### Statistical analysis

Statistical analyses were performed using IBM SPSS for Windows, Version 22.0 (IBM Corp., Armonk, NY, USA). Time to first therapy (TFT) and overall survival (OS) were analyzed on the date of the initial FISH study. Only leukemia-related deaths were considered when analyzing OS. The chi-square test was used to assess associations between categorical variables; continuous variables were analyzed with the Mann-Whitney *U* test. Variables significantly associated with TFT and OS were identified by the Kaplan-Meier method, and the curves of each group were compared with the log-rank test. Results were considered statistically significant for values of *p* < 0.05.

## Results

### Mutations of driver genes are already present in hematopoietic progenitor cells of CLL patients

**Fig. 1:**
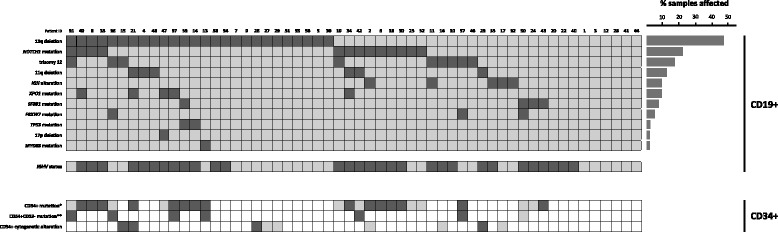
Cytogenetics and molecular characteristics of CD19+ and CD34+ cells. In the heatmap, *rows* correspond to the indicated alterations and each *column* represents individual CLL samples. Color-coded based on the gene and cytogenetic status (*dark gray*, altered; *light gray*, not-altered; *white*, not analyzed). For *IGHV* status: *dark gray*, unmutated; *light gray*, mutated. *Only mutations with VAF > 10% were considered for MACS isolated CD34+ cells considering the purities obtained. **Mutations in CD34+CD19− cells were assessed by ultra-deep NGS, considering mutations with VAF > 2%, taking into account the cell purities obtained from FACS sorting

Sequencing studies revealed a total of 28 mutations in 24 of the 56 (42.9%) CLL patients. Most of these patients (20/24; 83.3%) showed a single mutation in the analyzed genes, and four of them had two mutations in different genes (ID-34, ID-49, ID-50, and ID-53) (Table 1). The most frequently mutated gene was *NOTCH1* (23.2%), followed by *XPO1* (8.9%), *SF3B1* (7.1%), *FBXW7* (5.4%), *TP53* (3.6%), and *MYD88* (1.8%) (Fig. 1). All of them have been previously reported as mutations in the COSMIC database (http://cancer.sanger.ac.uk/cosmic). All patients with mutations in *NOTCH1* carried the same alteration (p.P2514Rfs*4), while *XPO1* mutations corresponded to a previously reported gain-of-function mutation (p.E571K) in all cases. In addition, all *SF3B1*, *FBXW7*, *TP53*, and *MYD88* mutations analyzed were missense mutations.

**Table 1:** Mutations in CD19+ and CD34+ cell populations identified in 56 CLL patients

ID	IGHV mutation status	Mutated gene	Exon	cDNA change	AA change	COSMIC ID	Mutational load CD19+ (%)	Presence in CD34+ cells^*^	Mutational load CD34+ (%)	CD19/CD34 ratio	Mutational pattern
2	Unmutated	*NOTCH1*	34	c.7541_7542delCT	p.P2514Rfs^*^4	COSM12774	51.0	Yes	31.0	1.6	Maintained
6	Unmutated	*NOTCH1*	34	c.7541_7542delCT	p.P2514Rfs^*^4	COSM12774	50.0	Yes	58.0	0.9	Maintained
8	Unmutated	*NOTCH1*	34	c.7541_7542delCT	p.P2514Rfs^*^4	COSM12774	97.5	Yes	79.5	1.2	Maintained
13	Mutated	*MYD88*	4	c.695T>C	p.M232T	COSM85942	34.0	Yes	22.5	1.5	Maintained
14	Unmutated	*TP53*	5	c.523C>G	p.R175G	COSM10870	87.5	Yes	12.0	7.3	Decreased
18	Unmutated	*NOTCH1*	34	c.7541_7542delCT	p.P2514Rfs^*^4	COSM12774	36.5	Yes	13.5	2.7	Decreased
19	Unmutated	*NOTCH1*	34	c.7541_7542delCT	p.P2514Rfs^*^4	COSM12774	10.0	No	< 10	–	–
24	Unmutated	*SF3B1*	16	c.2225G>A	p.G742D	COSM145923	24.0	No	< 10	> 2.5	Decreased
21	Unmutated	*XPO1*	15	c.1711G>A	p.E571K	COSM96797	39.5	Yes	31.0	1.3	Maintained
25	Mutated	*NOTCH1*	34	c.7541_7542delCT	p.P2514Rfs^*^4	COSM12774	3.0	No	< 10	–	–
30	Unmutated	*NOTCH1*	34	c.7541_7542delCT	p.P2514Rfs^*^4	COSM12774	57.0	Yes	24.0	2.4	Maintained
31	Mutated	*NOTCH1*	34	c.7541_7542delCT	p.P2514Rfs^*^4	COSM12774	4.5	No	< 10	–	–
33	Unmutated	*NOTCH1*	34	c.7541_7542delCT	p.P2514Rfs^*^4	COSM12774	41.0	Yes	40.5	1.0	Maintained
34	Unmutated	*NOTCH1*	34	c.7541_7542delCT	p.P2514Rfs^*^4	COSM12774	50.0	Yes	51.5	0.9	Maintained
34	Unmutated	*XPO1*	15	c.1711G>A	p.E571K	COSM96797	24.5	Yes	24.5	1.0	Maintained
36	Mutated	*FBXW7*	9	c.1394G>T	p.R465L	COSM33762	39.0	No	< 10	> 2.5	Decreased
37	Unmutated	*XPO1*	15	c.1711G>A	p.E571K	COSM96797	50.5	Yes	38.5	1.3	Maintained
42	Unmutated	*NOTCH1*	34	c.7541_7542delCT	p.P2514Rfs^*^4	COSM12774	5.0	No	< 10	–	–
43	Unmutated	*SF3B1*	15	c.2110A>T	p.I704F	COSM132954	47.5	Yes	45.0	1.1	Maintained
47	Unmutated	*XPO1*	15	c.1711G>A	p.E571K	COSM96797	2.5	No	< 10	–	–
49	Unmutated	*NOTCH1*	34	c.7541_7542delCT	p.P2514Rfs^*^4	COSM12774	55.0	Yes	56.0	0.9	Maintained
49	Unmutated	*XPO1*	15	c.1711G>A	p.E571K	COSM96797	42.5	Yes	31.5	1.4	Maintained
50	Unmutated	*FBXW7*	9	c.1268G>T	p.G423V	COSM1052095	8.0	No	< 10	–	–
50	Unmutated	*SF3B1*	14	c.1874G>A	p.R625H	COSM255276	21.0	No	< 10	> 2.5	Decreased
52	Mutated	*NOTCH1*	34	c.7541_7542delCT	p.P2514Rfs^*^4	COSM12774	16.0	No	< 10	> 2.5	Decreased
53	Unmutated	*SF3B1*	14	c.1996A>G	p.K666E	COSM110694	43.0	Yes	14.0	3.1	Decreased
53	Unmutated	*TP53*	7	c.734G>A	p.G245D	COSM43606	41.5	Yes	16.5	2.5	Decreased
57	Mutated	*FBXW7*	9	c.1394G>A	p.R465H	COSM22965	41.5	Yes	13.0	3.2	Decreased

^*^Only cases with > 10% CD34+ cells mutated were considered providing the purity for this cell fraction was higher than 90% in all the cases

**Fig. 2:**
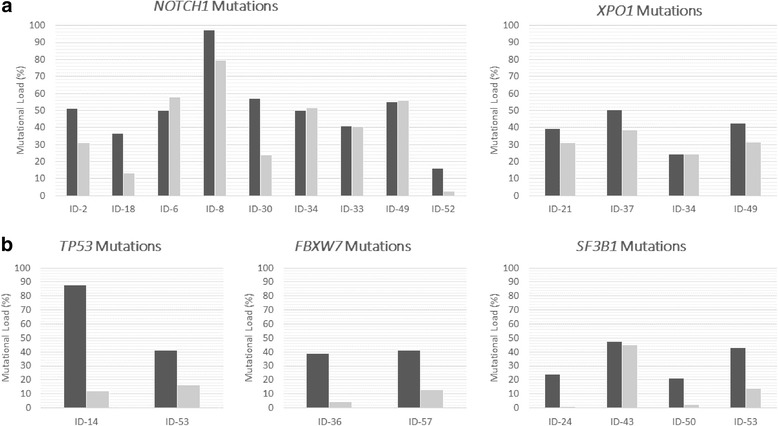
Mutational burden in CD19+ (*dark gray*) and CD34+ (*light* gray) cells from CLL patients. **a** *NOTCH1* and *XPO1* mutational burdens are similar in CD34+ and CD19+ cell populations. **b** *TP53*, *FBXW7*, and *SF3B1* mutational burdens were lower in the CD34+ cell population

In order to assess whether the mutations identified in CD19+ cells were also present in a previous step during B cell differentiation, the mutated target regions were analyzed by NGS in the total CD34+ cells. Strikingly, 15/24 patients (62.5%) showed the same mutations in their corresponding CD34+ cells (Table 1). The allele frequencies of mutations observed in a higher percentage than 10% of both CD19+ and CD34+ cell populations were compared calculating a CD19/CD34 ratio based on the percentage of mutated cells from each cell population. The cut-off CD19/CD34 ratio of 2.5 revealed two different mutational patterns between both cell fractions: “maintained” (ratio < 2.5) and “decreased” (ratio ≥ 2.5). Specifically, most of the mutations in *NOTCH1* (7/9) and *XPO1* (4/4) presented a similar mutational burden in both CD19+ and CD34+ cell fractions (Table 1; Fig. 2a). By contrast, alterations in *TP53* (2/2), *FBXW7* (2/2), and *SF3B1* (3/4) showed a clearly lower percentage or even an absence in the CD34+ cells with respect to the corresponding mature B lymphocytes (Table 1; Fig. 2b).

In a further step to assess if the mutations observed in the whole population of CD34+ progenitor cells appeared in the subpopulation of CD34+CD19− early hematopoietic progenitors, ultra-deep NGS was performed. Flow-sorted CD34+CD19− cells in a second time point from PB of a subset of patients were sequenced, confirming that six out of seven mutations—validated on the B lymphocytes from this time point—were also detected in the hematopoietic progenitor cells (Table 2). Particularly, *MYD88*, *NOTCH1*, *XPO1*, and *FBXW7* mutations were observed in CD34+CD19− cells. On the other hand, *SF3B1* mutation was not observed in the CD34+CD19− cells from patient ID-50. Apart from this, this patient, who was treated before the second time point, did not show *FBXW7* mutation in its B lymphocytes.

**Table 2:** Mutations in CD19+, CD34+CD19−, CD3+ and CD14+ PB cell populations identified by ultra-deep NGS

				Time point 1; bone narrow		Time point 2: peripheral blood			
Patient ID	*IGHV* mutation status	Mutated gene	AA change	%mut CD19	%mut CD34	%mut CD19+	%mut CD34+CD19−	%mut CD3+	%mut CD14+
13	Mutated	*MYD88*	p.M232T	36	22	**23.1**	**12.2**	**2.6**	**7.7**
31	Mutated	*NOTCH1*	p.P2514Rfs*4	4	< 10	**15.3**	**2.1**	0	0
36	Mutated	*FBXW7*	p.R465L	39	< 10	**3.2**	**3.0**	**2.7**	**3.6**
37	Unmutated	*XPO1*	p.E571K	51	38	**53.0**	**3.0**	**3.0**	0.4
42	Unmutated	*NOTCH1*	p.P2514Rfs*4	5.25	< 10	**33.0**	**12.9**	0	0.6
50	Unmutated	*SF3B1*	p.R625H	21	< 10	**15.0**	1.0	0.1	0.1
50	Unmutated	*FBXW7*	p.G423V	8	< 10	0	0	0	0
57	Mutated	*FBXW7*	p.R465H	42	13	**46.5**	**2.4**	0.8	**2.6**

The cut-off set for the second time point was 2% (bold) provided that our FACS purities were higher than 98% in all cases for all cell populations

### Distinctive pattern of distribution of CLL driver mutations along hematopoietic lineages

Ultra-deep NGS revealed that gene mutations can be also present in other mature cells derived from hematopoietic stem cells. Thus, the same mutations detected in CD19+ B lymphocytes as well as in their corresponding CD34+CD19− progenitors were also detected in a very low percentage of CD3+ cells and in CD14+ cells in some CLL patients (Table 2). Specifically, ID-13 and ID-36 patients, who harbored *MYD88* and *FBXW7* mutations, respectively, also presented these mutations in both CD3+ and CD14+ cell populations. Moreover, ID-57 (*FBXW7* mutated) showed the mutation on its monocytes whereas ID-37 (*XPO1* mutated) carried the same alteration on the T lymphocytes.

Interestingly, when the allele frequencies from all cell populations were compared, different patterns could be observed. First, *MYD88* and *NOTCH1* mutations (ID-13 and ID-42) appeared in > 10% of CD34+CD19− cells. In case of *XPO1* and *FBXW7*, their mutations also appeared in CD34+CD19− cells but in a relatively low percentage (< 5%). In addition, *NOTCH1* mutations only appeared on the hematopoietic progenitors and the mature B lymphocytes whereas *FBXW7* and *MYD88* mutations seemed to appear in all the sequenced cell fractions, affecting even myeloid lineage and T lymphoid lineage. On the other hand, patient ID-50 did not present its *SF3B1* mutation in the CD34+CD19− nor the T lymphocytes and monocytes (Table 2).

All these mutations detected at low frequencies (< 15%) in CD34+CD19−, CD3+, and CD14+ cells were also validated by 454 sequencing when material was available (Additional file 1: Table S3).

### Several cytogenetic abnormalities are detected in a previous developmental stage of the mature B lymphocytes of CLL patients

FISH studies revealed a total of 39/56 (69.6%) CLL patients with cytogenetic abnormalities in B lymphocytes. Specifically, 13q deletion was the most common aberration in our cohort (46.3%), followed by trisomy 12 (17%), 11q deletion (11.3%), *IGH* alterations (9.3%), and 17p deletion (1.9%) (Additional file 1: Table S1).

To asses if the chromosomal abnormalities where also present in a previous step of the B cell differentiation, FISH analyses were performed in the whole population of CD34+ cells of a subset of patients (*n* = 9) (Fig. 1). Interestingly, these analyses revealed that five of nine CLL patients with cytogenetic alterations in mature B lymphocytes showed the same chromosomal aberration in the CD34+ cells, although at a lower percentage than in CD19+ cells (Table 3). Specifically, both CLL patients with 11q- and three of five patients with 13q- showed the same cytogenetic alteration in the corresponding CD34+ cells. By contrast, *IGH* abnormalities identified by FISH in two CLL patients (ID-02 and ID-17) were not identified in their corresponding CD34+ cells. Apart from these alterations, the only CLL patient with +12, whose CD34+ cells were analyzed by FISH, did not have this trisomy in their corresponding progenitor cells.

**Table 3:** FISH analysis in CD34+ cell populations of nine patients

		FISH results				NGS results		
Patient ID	IGHV mutation status	Cytogenetic alteration	Altered CD19+ cells (%)	Presence in CD34+ cells	Altered CD34+ cells (%)	Mutated gene	Mutational load CD19+ (%)	Mutational load CD34+ (%)
2	Unmutated	*IGH alt*	80	No	–	*NOTCH1*	51	31
15	Mutated	13q-	65	Yes	25	–		
16	Unmutated	+12	22	No	–	–		
17	Mutated	*IGH* *alt*	54	No	–	–		
21	Unmutated	11q-	64	Yes	49	*XPO1*	39.5	31
21		13q-	93	Yes	72			
23	Unmutated	11q-	79	Yes	36	–		
26	Mutated	13q-	67	Yes	46.5	–		
27	Mutated	13q-	86	No	–	–		
29	Mutated	13q-	25	No	–	–		

### Patients with multiple genetic alterations show a hierarchy in the appearance of these events

The combination of NGS and FISH data revealed that two patients presented both mutations and chromosomal abnormalities (Table 3). The first case (ID-02) had an *IGH* alteration and a *NOTCH1* mutation in the CD19+ cells. However, FISH studies and NGS analysis of CD34+ progenitors revealed that the *NOTCH1* mutation was the only genetic event. The second case (ID-21) showed two chromosomal abnormalities (11q- and 13q-) and an *XPO1* mutation. These three alterations were also observed in the CD34+ cells, although 11q- and 13q- were present in a higher percentage of cells (72 and 49%) than the *XPO1* variant (30%).

Four cases showed a co-occurrence of mutations in two different genes. Thus, two patients (ID-34 and ID-49) carried mutations in *NOTCH1* and *XPO1*, present in both CD19+ and CD34+ cell fractions, with *NOTCH1* always being the dominant clone with respect to *XPO1* (52 vs. 25%; 55 vs. 31%). The patient ID-50 harbored mutations in *SF3B1* and *FBXW7*, which occurred at a much lower or null percentage in the CD34+ progenitors (21 vs. 2.5%; 8 vs. 0%). Finally, patient ID-53 had mutations in *SF3B1* and *TP53* in a low percentage of CD34+ cells compared with that in CD19+ B lymphocytes (43 vs. 14%; 41.5 vs. 16.5%).

### Clinical and biological correlations with genetic alterations in CD34+ cells in CLL patients

The clinical impact of the presence of mutations in the whole population of CD34+ cells was explored. The presence of mutations in the whole CD34+ cell population was associated with an unmutated *IGHV* status (*p* = 0.003) and high levels of serum β_2_ microglobulin (*p* = 0.011) (Additional file 1: Table S4). Interestingly, patients with mutations in their CD34+ progenitors also showed shorter OS (*p* = 0.01) (Additional file 1: Figure S2A) than patients without mutations in this cell fraction. In addition, patients harboring mutations in *NOTCH1*, *SF3B1*, and *TP53* in their hematopoietic progenitors presented a shorter TFT (*p* = 0.028) (Additional file 1: Figure S2B). Moreover, comparing the two mutational patterns identified in the CD34+ cells fraction, the mutations maintained on the CD34+ progenitors were significantly associated with the absence of trisomy 12 in the B lymphocytes (*p* = 0.014) (Additional file 1: Table S5).

Comparing the mutational burden in the CD34+ fraction between BM samples collected before and after treatment revealed no significant differences (*p* = 0.605). Four patients who received treatment before the extraction of the BM relapsed. Interestingly, all of them showed mutations in the CD34+ progenitors with a similar mutational burden as the corresponding CD19+ B lymphocytes (Additional file 1: Table S6).

## Discussion

Our data provide evidence of the presence of mutations and chromosomal abnormalities in early hematopoietic progenitors in BM samples of CLL patients. These results shed light on the cell of origin of CLL in a previous developmental stage to mature B cells, demonstrating CLL patients can also show genetic events in the CD34+ hematopoietic progenitors. These results are consistent with the findings of two recent studies [17, 18]. MACS isolation was performed on the whole population of CD34+ cells including CD34+CD19+ pro-B cells. A previous study has reported that the pro-B cell population is larger in the bone marrow of CLL patients than in healthy donors (mean range of 18% pro-B cells in the total bone marrow CD34+ cell count, exceeding 30% in some cases) [17]. This suggests that the patients who presented a mutational burden of < 30% in the CD34+ cell population may only harbor the mutation in CD34+CD19+ pro-B cells, rather than in the early hematopoietic progenitors. It is of particular note that 10 out of 15 patients with mutations in the whole population of CD34+ cells had mutational rates of > 30%, suggesting that these mutations not only appear in the pro-B cells, but also at earlier maturational stages of B cell differentiation (CD34+CD19− progenitors). In order to assess this hypothesis, we could perform ultra-deep NGS studies of flow-sorted CD34+CD19− cells using PB samples in a small subset of patients within the main cohort, detecting that all the mutations, except to one, were already present in this cellular fraction. Therefore, these results confirmed that mutations on CLL driver genes could occur in early hematopoietic progenitor cells of these patients. In particular, our sequencing results suggest that mutations in *NOTCH1*, *MYD88*, *FBXW7*, and *XPO1* may appear in CD34+CD19− cells whereas *TP53* and *SF3B1* mutations could appear in a later stage of B cell differentiation. As far as we are concerned, these results were demonstrated for the first time in fresh hematopoietic progenitor cells without having been cultured.

As these driver mutations have been detected in early hematopoietic progenitors of some CLL patients, we hypothesized that these alterations can affect hematopoietic lineages other than B cells. As it was previously reported, some mutations in well-known CLL drivers can also appear on a low percentage in other mature cell fractions as CD3+ T lymphocytes and/or CD14+ monocytes [18].

In order to determine the stage of B cell differentiation in which these mutations induced an expansion of the tumoral cell population, first, the mutational burdens in CD19+ mature B lymphocytes and CD34+ progenitors were compared. Since the mutational burden in CD34+ cells with *NOTCH1* mutations was as high as that observed in CD19+ cells in most of the cases, it could be hypothesized that mutations in this gene induced an expansion of the CLL hematopoietic progenitors. Indeed, ultra-deep NGS studies confirmed this in patient ID-42 who had *NOTCH1* mutation in more than 10% of CD34+CD19− cells. Moreover, every *XPO1* mutation observed in our cohort was present in the CD34+ progenitors and the mutational burden remained similar in both cellular fractions. However, the percentage of *XPO1* mutations in the CD34+ cells exceeded 30% in very few cases, suggesting that these mutations could be enriched at an intermediate B cell stage as CD34+CD19+ pro-B cells. On the other hand, the mutational burdens of *TP53*, *SF3B1*, and *FBXW7* were considerably lower in the CD34+ cell population. Although *SF3B1* mutation was not detected in CD34+CD19− cells, suggesting it as a late event in B-CLL differentiation, one out of four *SF3B1* mutated patients (ID-43) carried a mutation in a high percentage of the whole population of CD34+ progenitors, similar to results from a previous study [18]. Besides this, alterations in this gene have been also reported in CD34+ cells of patients with myeloid malignancies [33]. Therefore, it should be essential to study larger cohorts of CLL patients in order to determine what type of *SF3B1* mutations occur in HSCs of CLL patients and functional studies to assess the differences between “CLL-HSCs *SF3B1* mutated” and “MDS-HSCs *SF3B1* mutated.”

Focusing on the presence of cytogenetic abnormalities in CD34+ cell populations in our cohort, 11q- and 13q- appeared in the CD34+ progenitors at high percentages, as reported previously [15, 16], supporting the hypothesis that these chromosomal aberrations could be an early event in CLL [8]. By contrast, *IGH* alterations were not present in any of the CD34+ hematopoietic progenitors. Previous case report studies have yielded similar results [34, 35], suggesting that *IGH* alterations occur in an advanced stage of the lymphocyte maturation process.

**Fig. 3:**
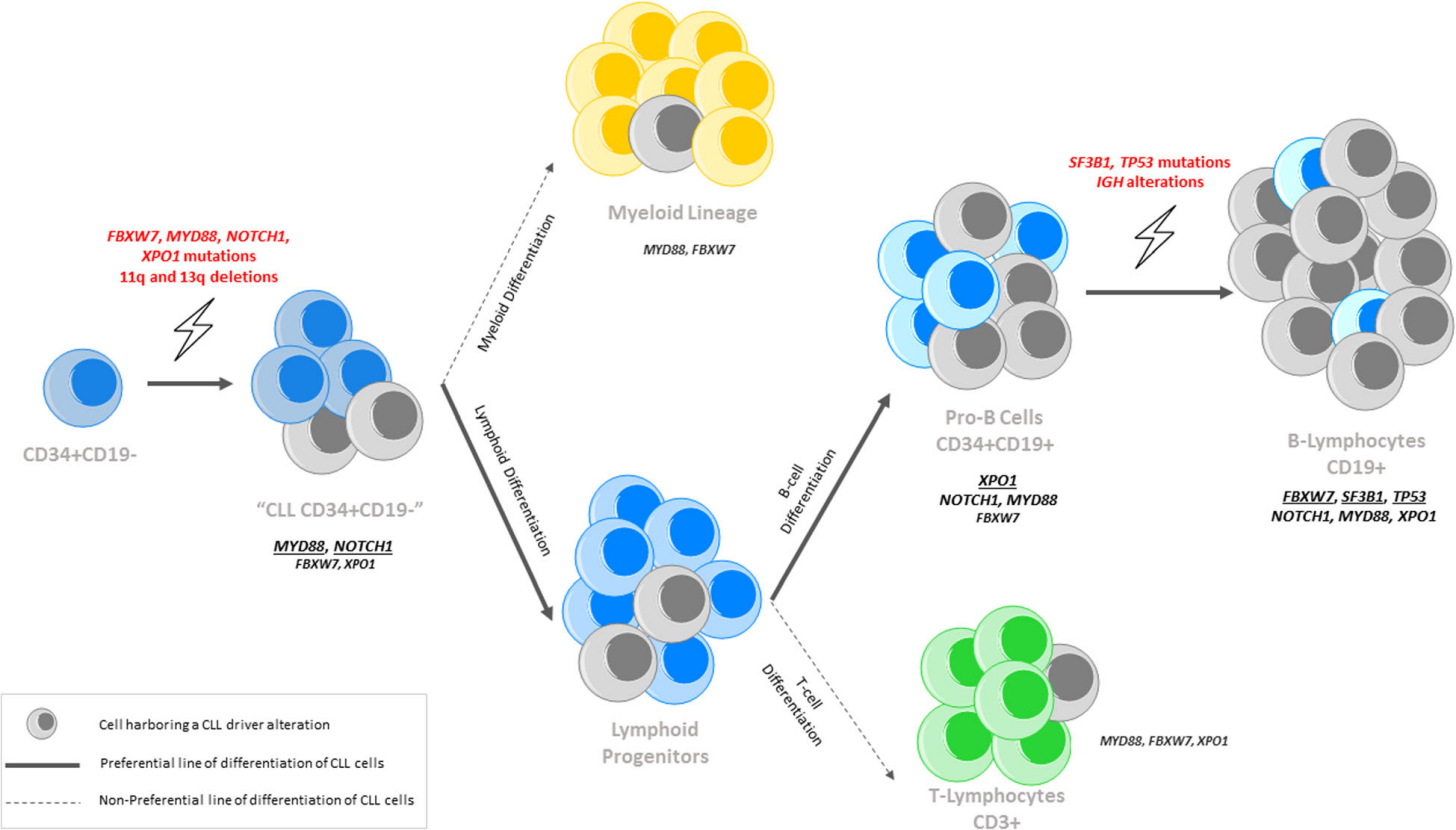
Schematic model of events in hematopoiesis in CLL patients. *Red gene* names indicate the moment of appearance of mutations. *Black names* indicate the presence of a gene mutation in an specific cell population whereas *underlined black gene* names indicate the moment of expansion of tumoral cells harboring these gene mutations during B-CLL differentiation

The analysis of patients with more than one genetic alteration allowed us to define a hierarchy of the appearance of these genetic events. When *IGH* alterations and the *NOTCH1* mutation are present in the same patient (ID-02), it is clear that the *NOTCH1* mutation is an earlier step than the *IGH* alteration during B cell differentiation. Moreover, when 11q-, 13q-, and *XPO1* mutations are present in the same patient (ID-21), they all appear in the CD34+ progenitors. However, 11q- and 13q- are certainly present at a higher allele frequency than the mutational load of *XPO1*, suggesting that cells with 11q- and 13q- were expanded in an earlier stage than the *XPO1* mutation in the pathogenesis of the disease [8]. Specifically, the dominant clone in the two cases with a double mutation in *NOTCH1* and *XPO1* (ID-34 and ID-49) was always *NOTCH1* rather than *XPO1.* Therefore, since *XPO1* is still present in a small part of the CD34+CD19− cell population and greatly enriched on the total CD34+ fraction, we may consider that cells carrying *XPO1* mutations are expanded in an intermediate event of B-CLL differentiation, given the previous possible events such as 13q- and 11q- abnormalities or *MYD88* and *NOTCH1* mutations. Taking into account all these results, a model of the appearance of genetic events during the hematopoiesis in CLL has been suggested (Fig. 3). However, as some of these genetic alterations were observed in few cases, it would be interesting to sequence all cell fractions in larger cohorts of patients.

The prognostic impact of gene mutations and chromosomal abnormalities in CLL has been well characterized in several studies of large cohorts of patients [4, 9, 23, 36]. *TP53*, *NOTCH1*, and *SF3B1* are described as poor prognostic mutations [10, 37–40]. The prognostic impact of the presence of mutations in CD34+ cells from CLL patients has been assessed for the first time in this study, showing that CLL patients harboring mutations in the hematopoietic progenitors showed worse prognosis. It is essential to achieve a better understanding of these results since only six genes were analyzed in our study, whereas a CLL exome exhibits an average of 20 mutations [8, 10]. Therefore, further studies analyzing the whole exome in larger cohorts should be performed in order to accurately define their impact. In addition, conventional CLL therapies may not be able to eradicate or reduce the malignant CD34+ CLL clone and may be responsible for the relapse of patients bearing mutations in these cells. Given that these therapies seem not to have a clear effect on CD34+ CLL cells, allogeneic stem cell transplantation could be an option for overcoming the challenges that may arise from CD34+ cell treatment [41].

## Conclusions

Our data show that recurrent CLL chromosomal abnormalities and gene mutations are present not only in mature B lymphocytes but also in hematopoietic progenitors. Although CLL is a clonal mature B cell disease, our results provide strong evidence that CLL may originate in the early stages of hematopoiesis. Both chromosomal alterations and point mutations are highly relevant to the disease pathogenesis, with a clinical impact as soon as they appear. To the best of our knowledge, our study is the first to analyze both genetic events in different cellular fractions of the same patients by NGS and FISH. It provides us with a broader understanding of CLL initiation and development, opening up possibilities for future therapies.

## Additional file

Additional file 1: Table S1. Clinico-biological characteristics from CLL patients. **Table S2.** Illumina Primer Design. **Table S3.** Validation of mutations detected at low frequency by ultra-deep NGS in flow-sorted cell fractions using 454 sequencing. **Table S4.** Patients’ characteristics regarding the presence of mutations in CD34+ progenitors. **Table S5.** Patients’ characteristics regarding the mutational burden maintenance or decrease in CD34+ progenitors. **Table S6.** Treatments prior bone marrow extraction of 4 CLL patients who relapsed and correlation with the CD19+ and CD34+ mutational status. **Figure S1.** Representation of the purity analysis of FACS sorted cell populations. **Figure S2.** Kaplan-Meier analysis of overall survival (A) and time to first therapy (B) in patients with mutations in their CD34+ progenitors. (DOCX 200 kb)

## Abbreviations

BM: Bone marrow
CLL: Chronic lymphocytic leukemia
FACS: Fluorescence-activated cell sorting
FISH: Fluorescence in situ hybridization
HSC: Hematopoietic stem cell
IGHV: Immunoglobulin heavy chain gene
MACS: Magnetically activated cell sorting
NGS: Next-generation sequencing
OS: Overall survival
PB: Peripheral blood
TFT: Time to first therapy
VAF: Variant allele frequency
