# Supplementary material for: Next-generation sequencing and FISH studies reveal the appearance of gene mutations and chromosomal abnormalities in hematopoietic progenitors in chronic lymphocytic leukemia
Source: J Hematol Oncol. 2017 Apr 11;10:83. doi: 10.1186/s13045-017-0450-y (PMC5387353; doi:10.1186/s13045-017-0450-y)
Supplement: Additional file 1: Table S1. — Clinico-biological characteristics from CLL patients. Table S2. Illumina Primer Design. Table S3. Validation of mutations detected at low frequency by ultra-deep NGS in flow-sorted cell fractions using 454 sequencing. Table S4. Patients’ characteristics regarding the presence of mutations in CD34+ progenitors. Table S5. Patients’ characteristics regarding the mutational burden maintenance or decrease in CD34+ progenitors. Table S6. Treatments prior bone marrow extraction of 4 CLL patients who relapsed and correlation with the CD19+ and CD34+ mutational status. Figure S1. Representation of the purity analysis of FACS sorted cell populations. Figure S2. Kaplan-Meier analysis of overall survival (A) and time to first therapy (B) in patients with mutations in their CD34+ progenitors. (DOCX 200 kb) [file 13045_2017_450_MOESM1_ESM.docx]

**ADDITIONAL FILES**

**Next-generation sequencing and FISH studies reveal the appearance of gene mutations and chromosomal abnormalities in hematopoietic progenitors in chronic lymphocytic leukemia**

Quijada-Álamo M, Hernández-Sánchez M et *al*.

The additional files consist on the following sections:

Additional Tables 1-6

Additional Figures 1-2

**ADDITIONAL TABLES**

**Additional Table 1. Clinico-biological characteristics from CLL patients.**

| **Characteristic** | **Category** |  |
| --- | --- | --- |
| **Age (years)** |  | 61 [25-91] |
| **Gender** | Male | 55.4% |
| **Binet Clinical Stage** | A | 70.3% |
|  | B | 27% |
|  | C | 2.7% |
| **Lymphadenopathies** | Yes | 51.4% |
| **Splenomegaly** | Yes | 15.8% |
| **Hepatomegaly** | Yes | 7.9% |
| **Leukocyte count (x10^9^/L)** |  | 16.7 [5.1-369] |
| **Lymphocyte count (x10^9^/L)** |  | 10.3 [1.5-355] |
| **Platelet count (x10^9^/L)** |  | 173 [83-337] |
| **Hemoglobin (g/dL)** |  | 14.1 [7.2-16.6] |
| **Serum LDH** | High | 3% |
| **Serum β_2_microglobulin** | High | 29.2% |
| ***IGHV* mutational status** | Unmutated | 53.6% |
| **CD38 expression** | Positive | 10.6% |
| **FISH** | Normal | 30.4% |
|  | 11q- | 11.3% |
|  | +12 | 17% |
|  | 13q- | 46.3% |
|  | *IGH* alterations | 9.3% |
|  | 17p- | 1.9% |
| **First Therapy*** | Yes | 84.2% |
| **Median TFT** (months)** |  | 35 |
| **Died during follow-up** | Yes | 11.4% |
| **Median OS*** (months)** |  | 76 |

*13 BM samples were collected after treatment.

**TFT: Time to first therapy; ***OS: Overall Survival.

**Additional Table 2. Illumina Primer Design.**

| **Gene** | **Exon** | **Forward Primer (5’-3’)** | **Reverse Primer (5’-3’)** | **Amplicon Product Length (bp)** |
| --- | --- | --- | --- | --- |
| *FBXW7* | 9 | GTGTTTTTCCAGTGTCTGAGAACAT | AGAAGTCCCAACCATGACAAGA | 227 |
| *MYD88* | 4 | TCCCAGGGGATATGCTGAAC | ATTCTCTTGCCAGAGCAGGG | 183 |
| *NOTCH1* | 34 | TGACCGCAGCCCAGTTC | ACTTGAAGGCCTCCGGAATG | 240 |
| *SF3B1* | 14 | TACCAACTCATGACTGTCCTTTC | CAGTGTGTCTCGCTTGCCA | 224 |
| *XPO1* | 15 | AGTAGGTCAATACCCACGTTTT | TCACAAGCCATATCCTGGACTC | 243 |

**Additional Table 3. Validation of mutations detected at low frequency by ultra-deep NGS in flow-sorted cell fractions using 454 sequencing.**

| **Patient ID** | **Mutated Gene** | **%mut CD34+CD19-** | **%mut CD3+** | **%mut CD14+** |
| --- | --- | --- | --- | --- |
| 13 | *MYD88* | **7.1** | 1.3 | **3.7** |
| 31 | *NOTCH1* | **2.7** | 0 | - |
| 37 | *XPO1* | - | **2.3** | - |
| 42 | *NOTCH1* | **5.1** | 0 | - |
| 50 | *SF3B1* | 1.6 | - | - |
| 57 | *FBXW7* | **4.4** | 0.9 | - |

**Additional Table 4**. **Patients characteristics regarding the presence of mutations in CD34+ progenitors**

| **Characteristic** | **Category** | **Patients with mutated CD34+ cells (n=15) (%)** | **Patients with unmutated CD34+ cells (n=41) (%)** | ***P*** |
| --- | --- | --- | --- | --- |
| **Age (years)** |  | 61 [50-83] | 61 [25-91] | 0.749 |
| **Leukocytes, range /µL** |  | 26200 [5090-369000] | 15900 [7200-279000] | 0.226 |
| **Lymphocytes, range /µL** |  | 20300 [1520-355000] | 9100 [3400-270000] | 0.226 |
| **Plateletcount, range /µL** |  | 137000 [83000-213000] | 176000 [95000-337000] | 0.416 |
| **Hemoglobin, range g/dL** |  | 12.8 [7.2-16.2] | 14.5 [8.0-16.6] | 0.08 |
| ***IGHV*** | Unmutated | 86.7 | 42.5 | **0.003** |
| **CD38** | Positive | 7.1 | 12.1 | 0.613 |
| **FISH** | **11q**- | 14.3 | 10.3 | 0.683 |
|  | **+12** | 7.1 | 20.5 | 0.253 |
|  | **13q-** | 53.3 | 43.6 | 0.520 |
|  | ***IGH* alt** | 7.1 | 10.0 | 0.751 |
|  | **17p-** | 0 | 2.6 | 0.545 |
| **Sex** | Male | 40.0 | 61.0 | 0.162 |
| **Β_2_microglobulin** | High | 62.5 | 12.5 | **0.011** |
| **Binet Stage** | B and C | 44.4 | 25.0 | 0.267 |
| **Lymphadenopathy** | Yes | 50.0 | 52.0 | 0.915 |
| **Hepatomegaly** | Yes | 20.0 | 3.6 | 0.098 |
| **Splenomegaly** | Yes | 30.0 | 10.7 | 0.151 |
| **Died during follow-up** | Yes | 23.1 | 0 | **0.007** |
| **Therapy during follow-up** | Yes | 90.9 | 80.8 | 0.444 |

**Additional Table 5**. **Patients characteristics regarding the mutational burden maintenance or decrease in CD34+ progenitors.**

| **Characteristic** | **Category** | **Mutations maintained on CD34+ progenitors (n=12)**  **(%)** | **Mutations decreased on CD34+ progenitors (n=8)**  **(%)** | ***P*** |
| --- | --- | --- | --- | --- |
| **Age (years)** |  | 60 [50-83] | 67 [60-82] | 0.302 |
| **Leukocytes, range /µL** |  | 18000 [5090-369000] | 26400 [11900-87300] | 1.0 |
| **Lymphocytes, range /µL** |  | 13900 [1520-355000] | 21100 [7200-74900] | 1.0 |
| **Platelet count, range /µL** |  | 150000 [100000-226000] | 116000 [83000-262000] | 1.0 |
| **Hemoglobin, range g/dL** |  | 12.6 [7.2-16.2] | 14.6 [8.0-15.3] | 1.0 |
| ***IGHV*** | Unmutated | 92.3 | 57.1 | 0.061 |
| **CD38** | Positive | 0 | 14.3 | 0.179 |
| **FISH** | **11q**- | 15.4 | 0 | 0.310 |
|  | **+12** | 0 | 33.3 | **0.028** |
|  | **13q-** | 46.2 | 42.9 | 0.888 |
|  | ***IGH* alt** | 15.4 | 0 | 0.310 |
|  | **17p-** | 0 | 0 | - |
| **Sex** | Male | 30.8 | 71.4 | 0.081 |
| **Β_2_microglobulin** | High | 50.0 | 100 | 0.197 |
| **Binet Stage** | B and C | 33.3 | 50.0 | 0.569 |
| **Lymphadenopathy** | Yes | 44.4 | 25.0 | 0.506 |
| **Hepatomegaly** | Yes | 22.2 | 0 | 0.255 |
| **Splenomegaly** | Yes | 22.2 | 20.0 | 0.923 |
| **Died during follow-up** | Yes | 27.3 | 33.3 | 0.793 |
| **Therapy during follow-up** | Yes | 90.0 | 100.0 | 0.464 |

**Additional Table 6. Treatments prior bone marrow extraction of 4 CLL patients who relapsed and correlation with the CD19+ and CD34+ mutational status.**

| **Patient ID** | **Last treatment before BM extraction** | **Time between last dose and BM extraction (months)** | **Mutated Gene** | **%CD19** | | **%CD34** | |
| --- | --- | --- | --- | --- | --- | --- | --- |
| 8 | Lenalidomide + Rituximab | 18 | *NOTCH1* | | 97.5 | | 79.5 |
| 33 | Fludarabine + Rituximab | 36 | *NOTCH1* | | 41.0 | | 40.5 |
| 34 | Bendamustine | 27 | *NOTCH1* | | 50.0 | | 51.5 |
|  |  |  | *XPO1* | | 24.5 | | 24.5 |
| 43 | Chlorambucil + Obinutuzumab | 31 | *SF3B1* | | 47.5 | | 45.0 |

**ADDITIONAL FIGURES**

**
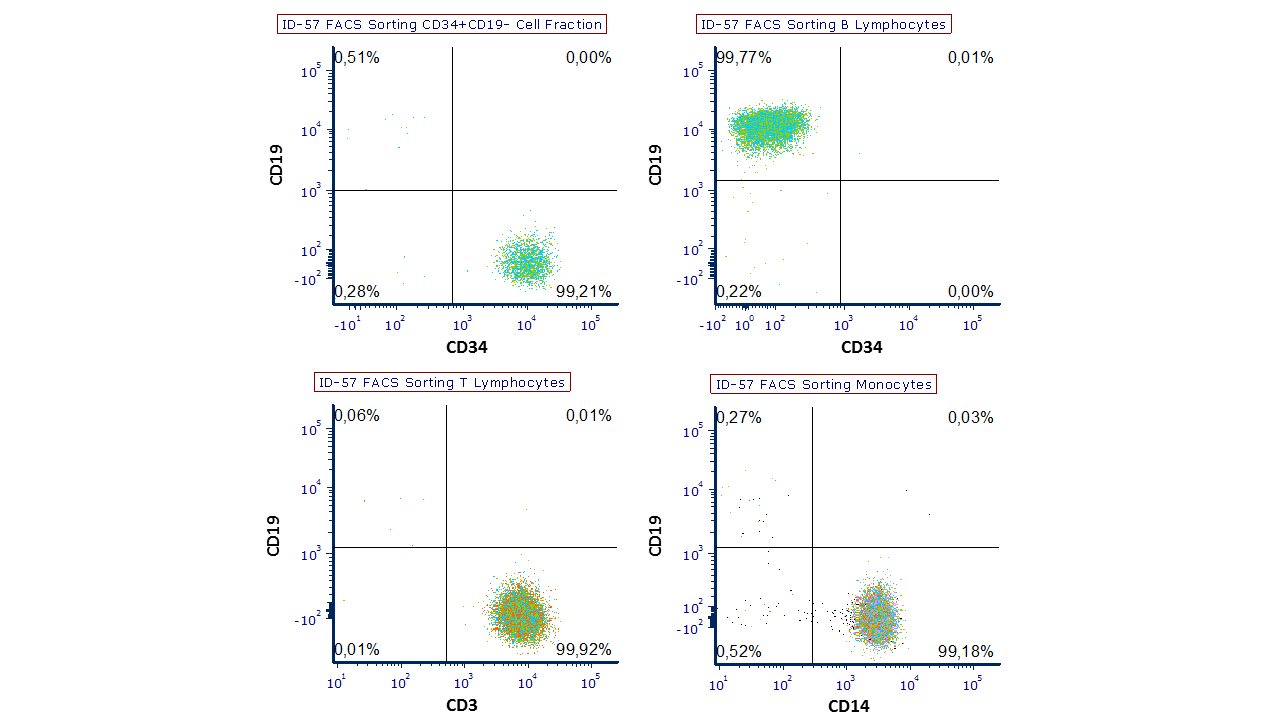
**

**Additional Figure 1. Representation of the purity analysis of FACS sorted cell populations.** CD34+CD19-hematopoietic stem cells; CD19+ B lymphocytes; CD3+ T lymphocytes; CD14+ monocytes.


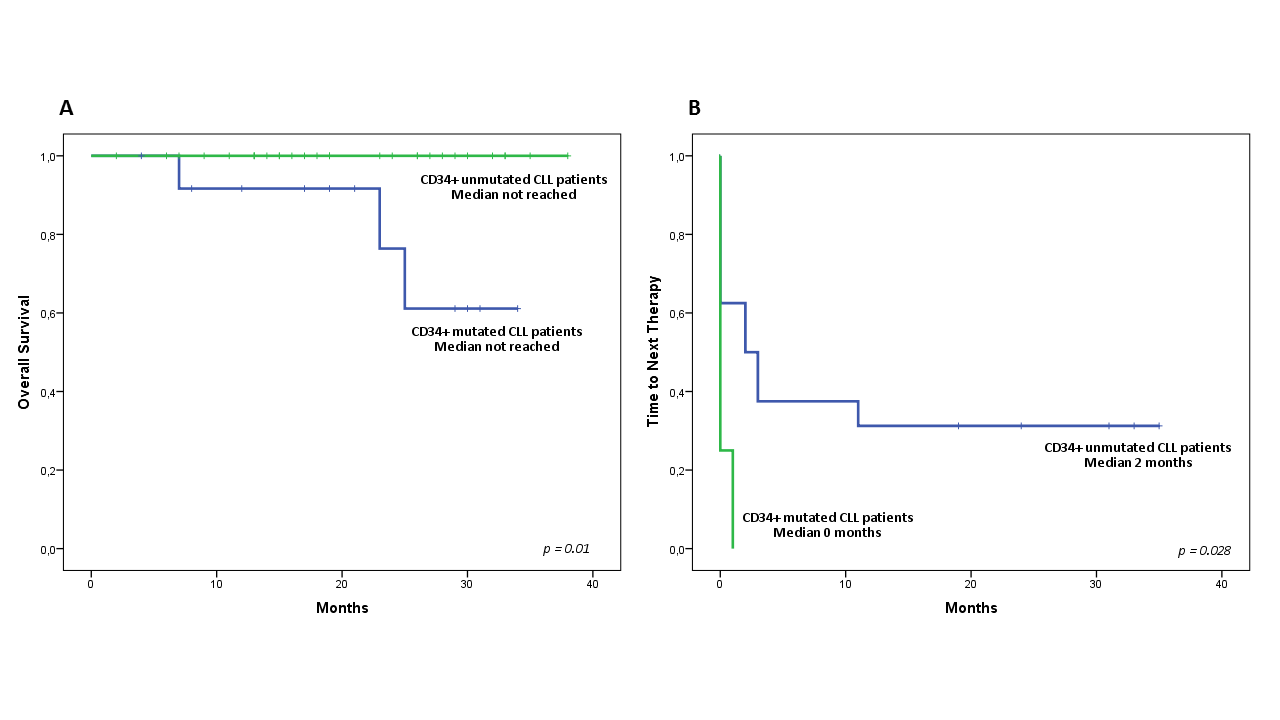


**Additional Figure 2**. **Kaplan-Meier analysis of overall survival (A) and time to first therapy (B) in patients with mutations in their CD34+ progenitors.**
